# Granzyme B in Inflammatory Diseases: Apoptosis, Inflammation, Extracellular Matrix Remodeling, Epithelial-to-Mesenchymal Transition and Fibrosis

**DOI:** 10.3389/fimmu.2020.587581

**Published:** 2020-11-11

**Authors:** Francesca Velotti, Ilaria Barchetta, Flavia Agata Cimini, Maria Gisella Cavallo

**Affiliations:** ^1^ Department of Ecological and Biological Sciences (DEB), Tuscia University, Viterbo, Italy; ^2^ Department of Experimental Medicine, Sapienza University of Rome, Rome, Italy

**Keywords:** granzyme B, inflammatory cytokines, inflammaging, extracellular matrix remodeling, anoikis, apoptosis, epithelial-to-mesenchymal transition, fibrosis

## Abstract

Inflammation is strictly interconnected to anti-inflammatory mechanisms to maintain tissue homeostasis. The disruption of immune homeostasis can lead to acute and chronic inflammatory diseases, as cardiovascular, pulmonary, metabolic diseases and cancer. The knowledge of the mechanisms involved in the development and progression of these pathological conditions is important to find effective therapies. Granzyme B (GrB) is a serine protease produced by a variety of immune, non-immune and tumor cells. Apoptotic intracellular and multiple extracellular functions of GrB have been recently identified. Its capability of cleaving extracellular matrix (ECM) components, cytokines, cell receptors and clotting proteins, revealed GrB as a potential multifunctional pro-inflammatory molecule with the capability of contributing to the pathogenesis of different inflammatory conditions, including inflammaging, acute and chronic inflammatory diseases and cancer. Here we give an overview of recent data concerning GrB activity on multiple targets, potentially allowing this enzyme to regulate a wide range of crucial biological processes that play a role in the development, progression and/or severity of inflammatory diseases. We focus our attention on the promotion by GrB of perforin-dependent and perforin-independent (anoikis) apoptosis, inflammation derived by the activation of some cytokines belonging to the IL-1 cytokine family, ECM remodeling, epithelial-to-mesenchymal transition (EMT) and fibrosis. A greater comprehension of the pathophysiological consequences of GrB-mediated multiple activities may favor the design of new therapies aim to inhibit different inflammatory pathological conditions such as inflammaging and age-related diseases, EMT and organ fibrosis.

## Introduction

Inflammation is a physiological response to infections or tissue injury and is essential for survival, having beneficial effects towards the neutralization of dangerous or harmful agents. Inflammation is strictly interconnected with anti-inflammatory mechanisms, which control and resolve the inflammatory process to maintain immune homeostasis (1). Under some circumstances, this immune homeostasis is disrupted and inflammation becomes excessive and/or persistent, leading to the development of inflammatory diseases (1). In this context, aging can be characterized by an uncontrolled and unresolved chronic, low-grade inflammation, the so-called ‘‘inflamm-aging”, which can lead to inflammatory age-related diseases, as cardiovascular, pulmonary, metabolic diseases (as type 2 diabetes, T2D) and cancer (2). Multiple factors underlie the pathogenesis of inflammatory diseases and can lead to tissue fibrosis and organ dysfunction, associated with high morbidity and mortality (3). Therefore, the knowledge of mechanisms involved in the development and/or progression of these pathological conditions is important to find specific and effective therapies.

Granzyme B (GrB) is a serine protease traditionally known for its perforin-dependent pro-apoptotic function underlying the capability of cytotoxic immune cells, as cytotoxic T lymphocytes (CTLs) and natural killer (NK) cells, to kill tumor and virus-infected target cells (4–7). GrB expression has been recently demonstrated also in non-tumor or tumor immune and non-immune cells (8). Indeed, GrB is produced and secreted by immune cells, like T and B cell subpopulations, monocyte/macrophages, mast cells, and basophils (8–13), by non-immune cells, like vascular smooth muscle cells (V-SMCs), pneumocytes, keratinocytes, and chondrocytes (12, 14–16), as well as by tumor cells, like leukemia cells and breast, urothelial, prostate, pancreatic and colorectal cancer cells (17–21) (**Table 1**). GrB not only exerts a perforin-dependent intracellular activity, but also an extracellular perforin-independent function, consisting in the cleavage of multiple extracellular substrates, as extracellular matrix (ECM) components, cytokines, cell receptors, angiogenic and clotting proteins (28, 49, 50). Hence, the pathophysiological function of GrB has been redefined and a putative role for GrB in the pathogenesis of inflammatory and age-related diseases has emerged (8, 29) (**Table 1**).

**Table 1:** GrB in Inflammatory Diseases: GrB producing cells, GrB cellular and molecular targets, GrB-associated organ-specific diseases.

GrB producing cells	GrB targets	GrB-associated organ-specific diseases
♦ Cytotoxic lymphocytes (4, 7) (CTL, NK cells) ♦ Non-cytotoxic immune cells (8–13) (monocytes/macrophages, B, T, granulocytes, mast cells, dendritic cells) ♦Non-immune cells (12, 14–16) (V-SMC, pneumocytes, keratinocytes, chondrocytes) ♦Tumor cells (17–21) (breast, urothelial, pancreatic, colorectal, prostate, leukemia)	♦ Normal Cells: -smooth muscle cells (14) -endothelial cells (14)	♦ Lung (22–27): COPD, RSV infection, pneumonia, IPF
	♦ Extracellular Molecules: -ECM proteins (28, 29) (fibrinogen, fibronectin, laminin, smooth muscle cell matrix, VE- cadherin, vitronectin, ZO-1) -ECM proteoglycans (30) (decorin, biglycan, soluble β-glycan) -IL-1 family cytokines (29, 31) (IL-1α, IL-18)	♦ Heart (32): cardiac fibrosis ♦ Adipose Tissue (33–35): adipose tissue fibrosis in metabolic diseases ♦ Blood vessels (8, 14, 36–48): atherosclerosis ♦ Skin (29, 49): skin fibrosis ♦ Breast, urothelial, pancreatic, colorectal carcinomas (18–21): invasion and EMT

CTL, cytotoxic T lymphocytes; NK, natural killer; V-SMC, vascular smooth muscle cells; ECM, extracellular matrix; VE, vascular endothelial; ZO-1, zonula occludens protein-1; IL, interleukin; COPD, chronic obstructive pulmonary disease; RSV, respiratory syncytial virus; IPF, idiopathic pulmonary fibrosis; EMT, epithelial-to-mesenchymal transition.

In this review, we discuss data concerning GrB activity on multiple targets involved in inflammation, potentially allowing this enzyme to regulate a wide range of crucial processes that play a role in inflammatory disease development, progression and severity. We focus our attention on the possible impact of GrB on inflammatory events leading to tissue fibrosis in both acute and age-related inflammatory diseases.

## Granzyme B as a Multi-Targeted Pro-inflammatory Molecule in Inflammatory Diseases

The recent discovery of multiple intracellular and extracellular substrates for GrB has revealed this protease as a potential multifunctional pro-inflammatory molecule, contributing to the pathogenesis of multiple pathological inflammatory conditions.

Elevated extracellular GrB levels were found in biological fluids, as in plasma from patients with acute myocardial infarction (36), atherosclerosis (37), obesity and T2D (51, 52), in broncho-alveolar lavage (BAL) in chronic obstructive pulmonary disease (COPD), pneumonia, and asthma (8), and in the synovial fluid in rheumatoid arthritis (53).

Elevated GrB levels were also found in inflamed tissues, including V-SMCs and atherosclerotic plaque in cardiovascular diseases (14), CTLs, pneumocytes and alveolar macrophages in pulmonary diseases (12, 27), adipose tissue-T cells in obesity (33) and in skin diseases (49). Moreover, according to a putative contribution of GrB in inflammaging, increased GrB expression levels were found in the elderly affected by obesity, cardiovascular and skin diseases (29, 49, 54, 55).

GrB extracellular substrates include cytokines and ECM components (29, 31, 50). The potential pathophysiological consequences of their cleavage constitute the basis to envisage a crucial pro-inflammatory role for GrB in the pathogenesis of inflammatory diseases (29).

GrB has the ability to process and activate pro-inflammatory, pro-fibrotic and aging mediators belonging to the IL-1 cytokine family (31, 56). Indeed, GrB processes IL-18 from its inactive to its active form and IL-1α into a significantly more potent pro-inflammatory fragment. IL-1α enhances persistent inflammation and stimulates fibroblasts to produce more interstitial collagenase and ECM remodeling, regulating normal and aberrant tissue repair (56, 57). IL-1α fragments, similar to those produced by GrB, were found in BAL in human airway inflammatory diseases, as COPD, cystic fibrosis and bronchiectasis (8), while GrB activity on IL-1α was demonstrated *in vivo* in GrB knockout mice (29), strongly suggesting that this activity also exists *in vivo*.

GrB has also the ability to degrade several ECM components, including proteins, as fibronectin, vitronectin, laminin, SMC matrix, VE-cadherin, and fibrillin-1, as well as proteoglycan, as biglycan and decorin, indicating GrB as a crucial player in ECM remodeling (28–30). Indeed, ECM undergoes remodeling, that is degradation by proteases and renewal and repair by fibroblasts, thus regulating tissue homeostasis and acting on tissue healing. ECM components assist cell attachment, ligate receptors and store growth factors, regulating cell survival, proliferation, differentiation, and migration. Therefore, abnormal ECM remodeling can result in cell detachment-dependent apoptosis and alterations in cell proliferation, differentiation and migration, as observed in several inflammatory conditions, such as cardiovascular, pulmonary and metabolic diseases, obesity, and cancer progression and metastasis (58, 59). Hence, GrB capability of targeting multiple ECM components, might allow this enzyme to regulate several fundamental biological processes involved in the development and/or progression of inflammatory diseases.

**Figure 1:**
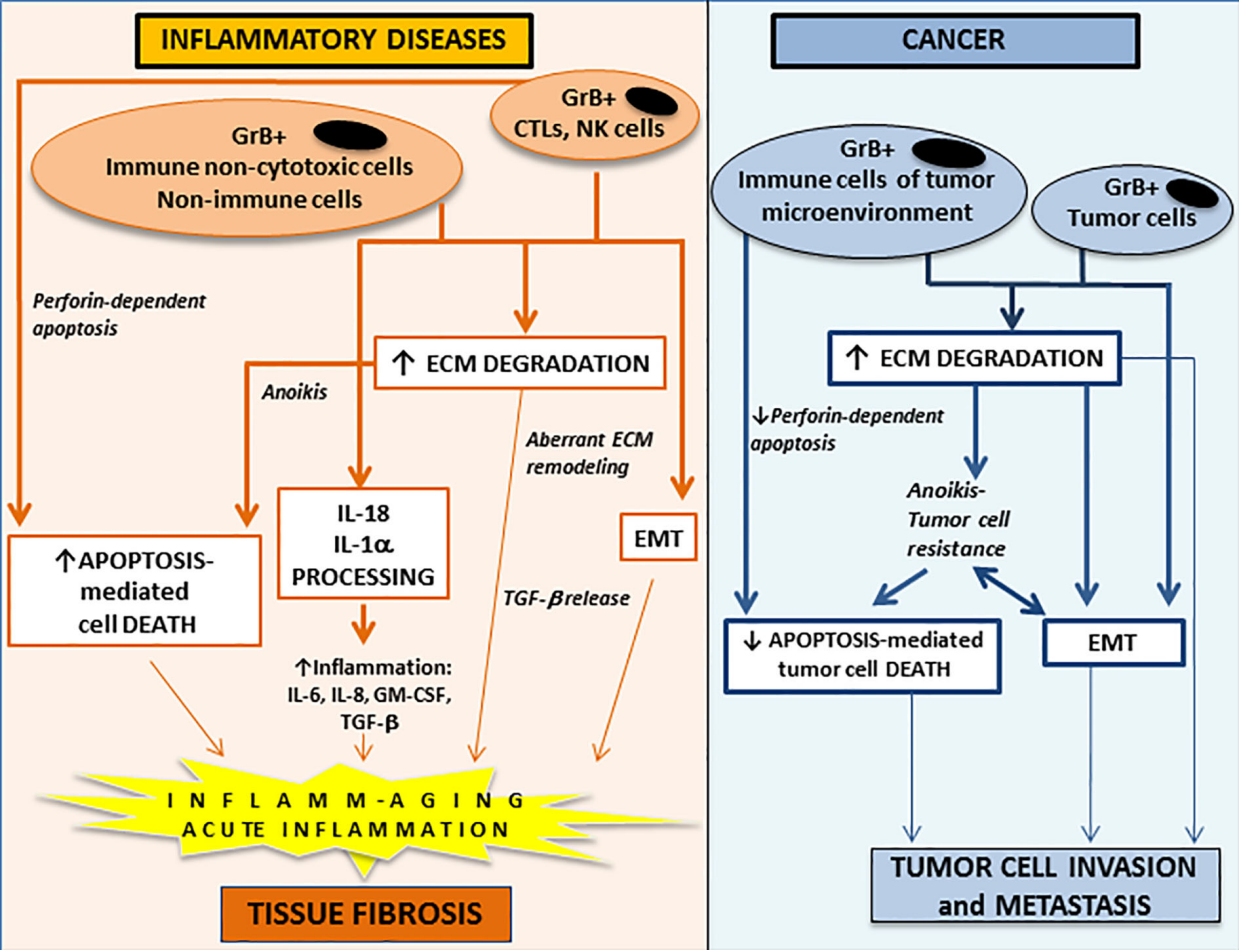
The potential contribution of extracellular and intracellular GrB functions to the development and/or the progression of acute and chronic inflammatory diseases (*left panel*) and to cancer invasion and metastasis (*right panel)*. GrB is a multifunctional pro-inflammatory molecule regulating a wide range of inflammatory events. GrB produced by perforin-expressing immune cells (CTL and NK cells) can induce perforin-dependent cell apoptosis, while GrB produced by perforin-deficient immune (e.g. non-cytotoxic T and B cell subpopulations, monocyte/macrophages/myeloid-derived suppressor cells, mast cells, basophils, neutrophils), non-immune (e.g. vascular smooth muscle cells, pneumocytes) and tumor (e.g. breast, urothelial, prostate, pancreatic, colorectal) cells can induce anoikis (anchorage-dependent cell death). Extracellular GrB can promote activation of pro-inflammatory cytokines (IL-18 and IL-1α), ECM degradation/remodeling, pathologic EMT and tissue fibrosis. GrB, granzyme B; CTL, cytotoxic T lymphocytes; NK, natural killer; ECM, extracellular matrix; EMT, epithelial-to-mesenchymal transition; IL, interleukin; TGF-β, transforming growth factor-β.

Thus, considering the extracellular and intracellular GrB function and the context in which GrB is produced, this molecule has the potential to contribute to the pathogenesis of non-neoplastic and neoplastic inflammatory diseases through a multitude of mechanisms ranging from the induction of perforin-dependent and/or –independent apoptosis and the promotion of epithelial-to-mesenchymal transition (EMT) and/or fibrosis, as illustrated below (**Figure 1**).

## Granzyme B and Perforin-Dependent and/or Perforin-Independent Apoptosis in Inflammatory Diseases

Apoptosis promotes tissue injury during inflammation and is involved in the pathogenesis of acute and chronic inflammatory diseases, as cardiovascular and pulmonary diseases and metabolic syndrome (60–62). The capability of immune and non-immune cell-derived GrB of inducing apoptosis makes GrB a potential important player of apoptosis-mediated tissue damage in inflammation. GrB can induce two kinds of apoptotic cell death, the intracellular perforin-dependent apoptosis (7) and the extracellular perforin-independent apoptosis, named anoikis (63). Anoikis is due to the detachment of cells from ECM and from neighboring cells, playing a role in preventing inappropriate cell translocation and attachment, and assisting appropriate tissue renewal (62). In cancer, anoikis resistance characterizes cancer cell anchorage-independent growth and EMT, contributing to cancer cell invasion and metastasis (62, 64, 65). In inflammatory diseases, as cardiovascular (66), pulmonary (67) and skin (49) diseases, and diabetes-related cardiovascular complications and retinopathy (62), aberrant anoikis is involved in excessive cell death and tissue injury.

A role for GrB-mediated apoptosis -either perforin-dependent apoptosis or anoikis- has been reported in inflammatory pulmonary diseases, including age-related diseases, as COPD (23, 38), and acute severe lung inflammatory diseases, as respiratory syncytial virus (RSV) pulmonary infections (24, 25). In COPD patients, GrB was identified in type II pneumocytes, alveolar macrophages and in bronchial and alveolar wall-infiltrating CTLs, suggesting a role for GrB in bronchial and alveolar cell apoptosis (8, 12, 22, 38). Of note, in COPD, increased GrB- and perforin-expressing CTLs were found in BAL and blood, and GrB-expressing T cells in BAL positively correlated with bronchial epithelial cell apoptosis (22, 23, 38). In spite of these findings, the evidence of a causative role for GrB-mediated apoptosis in the pathogenesis of COPD is lacking. *In vivo* animal studies are made difficult, because of the lack of appropriate mouse COPD models. A role for GrB has been proposed also in acute pulmonary pathologies. There is evidence of high GrB expression by CD8+T, CD4+T, and NK cells in human RSV-induced acute severe lung injury (24), suggesting a role for GrB in amplifying pro-apoptotic and pro-inflammatory activities. Supporting this hypothesis, Bem et al. (25) showed GrB contribution to acute lung injury in pneumovirus-infected mice; GrB deficiency in pneumovirus-infected mice significantly delayed clinical response to fatal pneumovirus infection and this effect was associated with delayed neutrophil recruitment, decreased caspase-3 activation and reduced lung permeability, suggesting a role for GrB in acute disease progression due to alveolar injury.

In the last years, a putative role for GrB-mediated apoptosis in atherosclerosis is also emerged in both the elderly and insulin resistant young individuals (8, 26, 39–41). Elevated plasma GrB levels were found in patients with myocardial infarction (36, 42) and unstable carotid plaques associated with increased cerebrovascular events (37). GrB was absent in normal vessels and its expression appeared during atherosclerosis; studies on mild and advanced atherosclerotic human coronary arteries showed higher GrB expression in V-SMCs, CTLs and macrophages in advanced lesions (11, 14). GrB expression co-localized with V-SMCs and macrophages undergoing apoptosis, suggesting that GrB may mediate apoptosis in these cells (11, 14). Furthermore, peripheral blood mononuclear cells (PBMCs) from patients with unstable angina produced higher GrB levels than PBMCs from patients with stable angina, and PBMC-derived conditioned media induced apoptosis in cultured endothelial cells, supporting a possible role for GrB in atherosclerosis severity, possibly inducing vascular apoptosis in unstable angina (43). Finally, the proteinase inhibitor-9, the GrB endogenous inhibitor, was reduced in unstable atherosclerotic lesions compared to stable lesions (44), according to the hypothesis of a role for GrB in plaque instability and suggesting that GrB activity in atherosclerosis may be regulated by an imbalance between GrB and its inhibitor. Although these findings do not allow to definitively establish an *in vivo* role for GrB in the induction of apoptosis in atherosclerotic plaque instability and rupture, *in vitro* and animal studies support this hypothesis (14, 26, 45, 46). Indeed, GrB mediates anoikis of cultured human coronary artery SMCs and endothelial cells (14). Moreover, in angiotensin II-treated apolipoprotein E (ApoE), GrB deficiency was associated with decreased abdominal aortic aneurysms and increased survival (because of rare aneurism rupture) compared to perforin-deficient or control mice (26, 45). Finally, a role for NK and NKT cells in the promotion of atherosclerosis has also been proposed (47, 48). Increased atherosclerosis was observed when NK cells were transferred into ApoE(-/-)Rag2(-/-)IL2rγ(-/-) mice, whereas decreased atherosclerotic lesions were found in NK cell depleted ApoE(-/-) or when GrB/perforin-deficient NK cells were transferred (47). Transfer of CD4+NKT cells into T-, B- and NK-cell-deficient ApoE mice augmented aortic root atherosclerosis; this effect reversed when GrB/perforin-deficient NKT cells were transferred (48).

## Granzyme B: Epithelial-to-Mesenchymal Transition and Fibrosis in Inflammatory Diseases

Inflammation, characterized by excessive apoptosis and abnormal ECM remodeling, can lead to tissue fibrosis, which impairs the affected organ’s function (3). Fibrosis is triggered by inflammatory cytokines and growth factors signaling abnormal ECM regulation; this leads to an imbalance between ECM degradation by proteases and excessive ECM deposition by different cells, mainly myofibroblast (3) derived by mesenchymal cells and by epithelial cells undergoing EMT (EMT-derived myofibroblasts) (68). Noteworthy, fibrosis and EMT share one of their major inducer that is transforming growth factor-*β* (TGF-*β*) (69).

Recent studies have proposed a role for GrB in heart, lung, adipose tissue and skin fibrosis (27, 29, 32, 49, 61, 69).

Elevated GrB expression was detected in human and murine fibrotic hearts (32). Moreover, a perforin-independent role for GrB in the pathogenesis of cardiac fibrosis was suggested *in vivo*, showing that GrB deficiency in mice protected against angiotensin II-induced cardiac fibrosis, reducing microhemorrhage, inflammation, and fibroblast accumulation (32).

In COPD, GrB–expressing monocytes and granulocytes were identified, and CD8+T infiltrating cells and apoptosis increased in airway epithelial cells, while soluble GrB levels and GrB-expressing T cells increased in BAL, suggesting that GrB upregulation in CD8+ and CD8- cells may be involved in small airway wall remodeling (27).

In obesity, increased CD8+T cells and GrB expression were found in adipose tissue *in vivo*, suggesting a role for GrB in adipose tissue fibrosis (33, 61). Furthermore, CD8+T-cell-depletion in overfed mice improved obesity-induced insulin resistance and decreased adipose tissue pro-inflammatory macrophages; these effects were reversed when mice were reconstituted with CD8+T cells (34). These findings suggest that, in obesity, adipose tissue CD8+T cells induce the recruitment of macrophages and that both may induce adipose tissue dysfunction and insulin resistance.

A role for GrB has also been indicated in skin fibrosis, as extensively discuss elsewhere (29, 49).

The mechanisms by which GrB induces fibrosis have not been completely elucidated and multiple GrB-mediated activities have been proposed.

GrB can cleave the ECM proteoglycan decorin, a potent anti-fibrotic (30) and a pro-autophagic (35) molecule. Indeed, decorin, by attaching to cell surface receptor and ECM molecules, regulates signal transduction pathways controlling genes involved in ECM organization (30). In addition, by attaching to cell receptors, decorin promotes autophagy in endothelial cells leading to inhibition of angiogenesis (35). Therefore, decorin cleavage by GrB might underlie aberrant ECM and/or vascular remodeling, involved in the initiation and/or the progression of various fibroproliferative disorders. Note also that decreased autophagy is involved in the pathogenesis of inflammaging, fibrotic diseases and tumors. Studies have shown a reduction of decorin in different fibrotic organs, as in cardiac fibrosis following myocardial infarction and in acute exacerbation-idiopathic pulmonary fibrosis (IPF) (70–72). Animal experiments in decorin-null mice with myocardial infarction (70) or in hamster and mice models of lung fibrosis (73–75) showed both decorin requirement for proper fibrotic evolution of tissue injury and the potential therapeutic anti-fibrotic effect of decorin administration. Evidence also exists for a role of decorin in maintaining glucose tolerance in obesity (76).

Moreover, GrB, cleaving decorin and other ECM substrates as biglycan, beta-glycan and fibrillin-1 which act as reservoir of cytokines and growth factors as TGF-β, induces the release of active TGF-β, a key regulator of fibrosis (30, 69). Therefore, the aberrant release of sequestered TGF-β by GrB-mediated cleavage of ECM components represents another potential mechanism by which GrB may contribute to fibrosis.

Noteworthy, GrB (18, 21, 77), as some other granzymes (78–80), has been recently proposed as promoters of EMT, an important process linked the stimulation of the three following events: 1) tissue and organ formation during embryogenesis; 2) tissue and organ physiologic repair and pathologic fibrosis; 3) tumor cell invasion and metastasis (81). EMT is a process in which epithelial cells lose E-cadherin-mediated cell-cell adhesion and acquire some mesenchymal features, as N-cadherin expression and the capability of invasion, migration, and production of ECM. Inflammatory molecules, mainly TGF-β, trigger intracellular signaling cascades, activating EMT-transcription factors like Snail, ZEB, and TWIST (81). EMT is involved in multiple organ fibrosis, as those occurring in cardiovascular and pulmonary (COPD and IPF) diseases (81–88). Now interestingly, EMT-derived fibrosis has been also called to possibly account to pulmonary fibrosis in SARS-CoV-2 infection (89), suggesting a possible contribution of GrB in the severe pulmonary damage in COVID-19 (89, 90). A possible role for GrB in EMT promotion has emerged in human tumor models (18, 21, 77). Enzymatically active GrB was expressed, in absence of perforin, by tumor cells *in vitro* and in tissues (*ex vivo*) (17–21). Although GrB in cancer tissues is widely used as activation marker for cytotoxic lymphocytes, and lymphocyte-derived GrB-positive tumor immunostaining is associated with a favorable clinical outcome in a large spectrum of cancers, in some cases, GrB expression in tumors correlates to the severity of the disease, poor prognosis and therapy resistance (91–96). It has been documented GrB expression by urothelial carcinoma cells in primary urothelial cancer tissues and its expression was associated to EMT (analyzed by Snail-1, E- and N-cadherin expression) (18). Significantly, GrB expression was concentrated in urothelial neoplastic cells undergoing EMT at the cancer invasion front, suggesting that the expression of GrB and EMT molecules might be functionally related (18). A further support to the hypothesis of considering GrB as an EMT promoter, derives from the association that existed between GrB expression in tumor tissues and the pathological tumor spreading, in particular, the increasing invasiveness status of urothelial carcinomas (18). In addition, *in vitro* experiments of loss and gain of GrB function performed in CRC (including also CRC patient-derived Cancer Stem Cells), bladder and pancreatic carcinoma cells showed that GrB deficiency was associated to the loss of the EMT phenotype and the inhibition of invasion through matrigel, further supporting a role for GrB in tumor EMT promotion and cancer cell invasion (18, 21). Finally, GrB function in EMT was further supported by data indicating a contribution of GrB in the induction of TGF-β1-driven EMT in CRC cells (21). Indeed, TGF-β1 enhanced GrB expression while inducing EMT in CRC cells, whereas GrB depletion resulted in the inhibition of TGF-β1-driven EMT (21). However, research is needed to identify GrB targets involved in the mechanisms underlying EMT modulation by GrB. It should also be taken into account the possible regulation of GrB activity and function by the GrB-bound proteoglycan serglycin, considering that its intracellular activity consists in the promotion of secretory granule maturation and GrB storage, while its extracellular activity is implicated in the regulation of tumorigenesis, driving inflammation, EMT and tumor progression (97). Lastly, the examination of GrB expression in a large number of cancers in relation to the clinical outcome is needed, together with the evaluation of EMT in murine tumor and non-tumor models.

## Conclusion

GrB is emerging as a multifunctional pro-inflammatory protease, acting with tissue and context dependence on multiple targets, thus representing a putative powerful regulator of a wide range of crucial processes involved in the pathogenicity and/or in the severity of inflammatory diseases, either acute or age-related. The major limitation of this assumption is the paucity of *in vivo* direct evidence for the multiple GrB pro-inflammatory activities. It should be considered that the *in vivo* function of human GrB is a challenging problem and difficult to deal with, in that, although few mechanistic animal studies connecting clinical observations with *in vitro* data exist, animal experiments might generate false interspecies functions of GrB, because of GrB interspecies structural and functional diversity (98–100). Therefore, further research is required to explore the multiple activities of GrB potentially occurring in the inflammatory events underlying acute and chronic inflammatory diseases.

A greater comprehension of GrB function may favor the design of new therapies aimed to inhibit and regulate GrB pro-inflammatory activities, counteracting excessive inflammation, fibrosis and abnormal EMT-derived processes. Current research is considering the development and the use of pharmacological GrB inhibitors as potential therapeutic options for the prevention and/or treatment of GrB-associated inflammatory pathological conditions (101–106). Progress in this field might be even more urgent if we consider the possibility to develop therapies that have an impact on inflammaging and chronic age-related diseases, as well as on excessive acute inflammatory reactions, as they occur in COVID-19, especially in aged individuals tending to excessive inflammatory responses resulting in lethal lung damage (89, 107, 108).

## Author Contributions

FV made substantial contributions to conception and design of the review. IB, FAC, and MGC contributed to the manuscript revision, read, and approved the submitted version. All authors contributed to the article and approved the submitted version.

## Funding

IB is supported by a research fellowship from Eli Lilly Foundation. FAC was supported by research funding from Sapienza University, Rome, Italy. This work was supported by research funding from Department of Experimental Medicine (Rome, Italy) to MGC.

## Conflict of Interest

The authors declare that the research was conducted in the absence of any commercial or financial relationships that could be construed as a potential conflict of interest.

The handling editor declared a shared affiliation with several of the authors IB, FAC, MGC at time of review.
